# Supplementary material for: Application of Immersive Virtual-Reality-Based Puzzle Games in Elderly Patients with Post-Stroke Cognitive Impairment: A Pilot Study
Source: Brain Sci. 2022 Dec 31;13(1):79. doi: 10.3390/brainsci13010079 (PMC9856594; doi:10.3390/brainsci13010079)
Supplement: Supplementary file 1 [file brainsci-13-00079-s001.zip › Supplementary materials.pdf]

Table S1. The self-report questionnaire associated with IVR

Parts	Items	
I. Experience with using smart devices	3	Previous experience using smart devices.
II. VR equipment and training content experience	6	Comprehension, enjoyment, acceptability, stability, maneuverability, recommendation
III. Adverse reactions	5	Dizziness, nausea, headache, dry eyes, eye strain.
